# Promoting successful participation of people living with Alzheimer's disease and related dementias in pain-related neuroimaging research studies

**DOI:** 10.3389/fpain.2022.926459

**Published:** 2022-08-19

**Authors:** Wm. Larkin Iversen, Todd B. Monroe, Sebastian Atalla, Alison R. Anderson, Ronald L. Cowan, Kathy D. Wright, Michelle D. Failla, Karen O. Moss

**Affiliations:** ^1^College of Nursing, The Ohio State University, Columbus, OH, United States; ^2^Department of Physics and Astronomy, University of North Carolina at Chapel Hill, Chapel Hill, NC, United States; ^3^University of Tennessee Health Science Center, Memphis, TN, United States

**Keywords:** ethics, neuroimaging, recruitment, dementia—Alzheimer's disease, consent (incapable adults)

## Abstract

Recruitment and retention of participants for pain-related neuroimaging research is challenging and becomes increasingly so when research participants have a diagnosis of Alzheimer's disease and related dementias (ADRD). This article shares the authors' recommendations from several years of successful recruitment and completion of pain-related neuroimaging studies of people living with ADRD and includes supportive literature. While not an exhaustive list, this review covers several topics related to recruitment and retention of participants living with ADRD, including community engagement, capacity to consent, dementia diagnostic criteria, pain medication and other study exclusion criteria, participant and caregiver burden, communication concerns, and relationships with neuroimaging facilities. Threaded throughout the paper are important cultural considerations. Additionally, we discuss implications of the coronavirus (COVID-19) pandemic for recruitment. Once tailored to specific research study protocols, these proven strategies may assist researchers with successfully recruiting and retaining participants living with ADRD for pain-related neuroimaging research studies toward improving overall health outcomes.

Recruitment and retention of research participants constitutes one of the most important but challenging aspects of clinical research. Complex protocols and procedures further increase this difficulty, lessening assurance of successful study completion. Some populations, such as people living with Alzheimer's disease and related dementias (ADRD), present specific challenges for researchers investigating clinically meaningful questions that are reliant on neuroimaging procedures. While these Institutional Review Board (IRB)-approved health-related procedures are proven safe, neuroimaging involving people living with ADRD requires unique approaches, given their altered cognitive and communication abilities that are often combined with physical health and other challenges. Cognitive impairment may diminish participants' decision-making capacity as well as their ability to communicate with research personnel without the assistance of a surrogate decision maker/family caregiver. Given these challenges, researchers may need to recruit and retain both a study participant and their family caregiver as a study partner. In addition, many people with declining cognitive skills reside in congregate living facilities, which introduces privacy concerns that may hinder recruitment efforts (1). Thus, issues regarding decisional capacity and privacy concerns arising from place of residence can significantly hinder the recruitment of participants living with ADRD and its associated cognitive challenges (2–4).

Unfortunately, literature pertaining to effective recruitment and retention methods in populations living with ADRD is sparse, particularly in regard to pragmatic solutions to commonly identified recruitment barriers. While the Alzheimer's Disease Neuroimaging Initiative (ADNI) has resulted in many studies that have advanced our understanding of ADRD, researchers often face challenges with recruitment and retention of participants for neuroimaging research studies (5–7). Additionally, pain was introduced as the fifth vital sign in 1995 (8) and the importance of identifying and addressing pain in ADRD cannot be overstated. This added focus on pain-related research, in this population introduces the potential for additional research-related challenges (9–11). In this article, we share our extensive experience from multiple studies conducted across several large academic medical centers and lessons learned in successfully recruiting these participants and their families. We will address the following topics: community engagement, capacity to consent, dementia diagnostic criteria, pain medication and other exclusion criteria, participant and caregiver burden, communication concerns, relationship with neuroimaging (specifically magnetic resonance imaging (MRI) facilities, and the implications of the Coronavirus Disease 2019 (COVID-19) pandemic on recruitment and retention. In addition, we provide possible solutions, and as appropriate, supplement our discussion with supporting research literature.

## Community engagement

### Barrier

At the very start of a study, researchers may have difficulty recruiting study participants, given the sometimes inaccessible nature of or restricted access to their place of residence (e.g., long-term care facility). Some individuals living with ADRD are no longer sufficiently mobile enough or cognitively able to safely navigate driving a vehicle. Some may also lack access to reliable transportation in the form of a trusted family member or friend (a caregiver) who is capable of driving and has access to a vehicle to drive them to a neuroimaging facility. Additionally, costs for car gasoline, taxi, and other ride share services to and from a neuroimaging facility may also present a barrier for these older adult study participants.

### Solution

Our experience is that studies with an ADRD population will be strengthened if the research team seeks, develops, and maintains community partnerships with organizations such as adult daycare centers, senior service providers, assisted living facilities, and nursing homes *via* regular emails and face-to-face meetings (2, 3). Fostering personal relationships between research personnel and community ambassadors, including family caregivers, support groups, and medical professionals has resulted in establishing a robust network for community engagement. Distributing fliers, pamphlets, posters, brochures, or letters to the directors of such facilities or affiliated physicians, such as gerontologists, is cost- and time-efficient, as is word-of-mouth which yields a high degree of study exposure. Because of the word-of-mouth nature of exposure, it is important to ensure that positive impressions are made. Establishing and maintaining trust between researchers and community members living with ADRD, family and professional caregivers, as well as organization leaders is critical (2, 3). This includes addressing culturally-specific barriers that includes, but is not limited to those such as language through the provision of translation, and use of instruments already translated into the preferred language that is best understood by participants. Facility directors, physicians, nurses, and other healthcare providers can serve as ambassadors who help to establish and maintain trust, particularly as it relates to recruiting and retaining individuals from under-represented racial/ethnic, socioeconomic status and gender groups. Such collaborations creates an invaluable network that provides reliable access to potential participants for current and future research recruitment. Financial compensation of participants and their caregivers not only increases interest and commitment but may lend additional credibility to the research project as a demonstration of the value and respect for participants' time commitment. Our studies often include other data collection components such as survey and psychophysical data collection in addition to neuroimaging. Therefore, the value of compensation is weighed against participant time for engagement, cost for transportation (to and from data collection site), burden, etc. and payment ranges from $150 to $300 in total depending on which study aspect(s) the participant and caregiver completes. We recognize that caregivers play a significant role in successful recruitment and retention due to their time commitment. Both financial or non-financial compensation to organizations that assist with recruitment should also be considered. This may include delivery of expert in-person or virtual scientific presentations to healthcare staff (providing continuing education credits, if possible) or presentations to lay individuals, such as, those living with early stage ADRD and caregiver support group members.

Many communities have agencies and centers for caregivers of those living with ADRD, ranging from clinics to outreach programs to support groups. Coordinating with these centers can be invaluable for recruitment as a site for garnering “snowball” referrals (a technique whereby a participant's friends and family are asked to identify other possible participants) (12, 13). Caregivers' lives are heavily impacted by their loved one's disease, and they may be willing to contribute in this meaningful way to scientific knowledge about ADRD (4). Participants and caregivers have shared that they find engaging in research activities comforting, as it demonstrates that a concerted effort is underway to better understand the disease (14–16).

Two final recommendations: First, we urge researchers to make the task of securing a sufficient number of dedicated study recruitment personnel with recruitment expertise is a top priority. To help determine the number of personnel needed to successfully complete a study, we conducted a program analysis aimed to identify recruitment outcomes associated with a dedicated staff member. Over a period of approximately 5 years of active recruitment (prior to and during the COVID-19 pandemic), a specialist and a full-time research assistant screened and enrolled an average of eight participants (∑ = 8.16) living with ADRD per month; two of eight monthly enrollees (∑ = 1.97) successfully completed neuroimaging (17). Research projects requiring a larger data set will need additional staff and funding to achieve the goals of the study. Therefore, we suggest funding allocations for this purpose as well as advertising costs be included and re-evaluated as needed overtime.

It is important to note that because of traumas stemming from historical atrocities such as slavery, as well as ongoing systemic racism and discrimination in medicine, members of underrepresented racial and ethnic groups may not be inclined to participate in medical research. For example, the unethical treatment of African Americans in the Tuskegee Syphilis Study significantly impacts distrust of researchers, healthcare institutions, and providers that continues today (18, 19). Hiring research staff from within subcommunities of interest or who reflect its demographics (12), together with the use of snowball referrals, helps to increase trust and acceptance for such medical research by underrepresented racial/ethnic groups living with ADRD (20). Further, having a diverse research team representative of the various communities in which we live is of extreme importance and can ensure representation of valuable insights on ADRD impacts across racial and ethnic groups (19). This is a key step toward ensuring health equity (19), a goal to which all researchers must be committed.

## Capacity to consent

### Barrier

People living with ADRD may have diminished capacity to provide the requisite informed consent to participate in research. Yet the investigative team has a duty to assess and determine each participant's capacity to provide informed consent/assent once the initial screening requirements to be included in a study have been determined (9).

### Solution

There are multiple tools available to determine an individual's capacity to provide consent for research participation. One example that is widely used is the MacArthur Competence Assessment Tool for Clinical Research (MacCAT-CR) (21, 22). The MacCAT-CR is a 21-item questionnaire with four subscales that assess understanding, appreciation, reasoning, and expression of choice and take approximately 20 min to complete (21). The questions are tailored to the specific research context and instead of a threshold or limit score, the assessor uses it as guide for determining decision making capacity (21). The MacCAT-CR has a high interrater reliability and was found to be feasible for use (22).

Another such screening instrument is the University of California-San Diego's Brief Assessment of Capacity to Consent (UBACC). This 20-item questionnaire takes approximately 5 min to complete, and psychometric testing has shown that scores > 14.5 were 89% sensitive and 100% specific for determining capacity to consent for research (23). Since determining that a potential participant's diagnosis of ADRD or dementia alone is not sufficient to assess capacity, a tool such as the UBACC can be administered to any participant other than a person without cognitive impairment in order to meet the duty to assure consent. If possible, participants who score <14.5 on the UBACC should sign an assent document. In addition, surrogate consent is generally required from a caregiver or legal guardian to meet the duty of consent. Use of the UBACC screening study in our study on ADRD and pain helped us determine who had the capacity to self-consent relative to those who required caregiver and surrogate assent (10, 11).

Potential alternatives to the MacCAT-CR and UBACC is the use of a three-item decisional capacity to consent questionnaire (24). A version of such a questionnaire is described by Palmer and colleagues and was found to be sensitive to impaired understanding when compared with the MacCAT-CR (24). The three-item capacity questionnaire described by Palmer and colleagues consists if the following questions: (1) “What is the purpose of the study?” (2) “What are the risks?” and (3) “What are the benefits?” (24) Similarly, Monroe and colleagues uses the following 3 questions to assess decisional capacity to consent: (1) “Can you name one thing that will happen if you participate in the study?” (2) “What can you do to withdraw from the study?” and (3) “What types of questions will we ask you as part of this study?” (25) When using the three-item capacity to consent questionnaire, participants with cognitive impairment who correctly answer all 3 question prompts will be permitted to sign the informed consent document; those incorrectly answering any question or prompt on the three-item capacity to consent questionnaire will be permitted to sign the assent document and surrogate consent obtained. Using a three-item capacity to consent questionnaire in our study on dementia and pain in the nursing home helped us correctly determine the ability of participants (~50%) to self-consent while excluding those unable to self-consent (25). Use of a 3-item capacity to consent questionnaire has not been widely cited as a standardized evaluation criteria, however, based on our experiences in past (25) ongoing (26) and other studies (24) we highly recommend its use in conjunction with consent and assent documents as applicable for this purpose.

## Dementia diagnostic criteria

### Barrier

Some research programs rather loosely define what is meant by *dementia*, since it is a broad term that includes any dementia subtype (e.g., Alzheimer's disease, vascular dementia, Lewy body dementia) or even comorbidities that may induce cognitive decline, such as Pick's disease or Parkinson's disease (27, 28). Further, the investigative team may not have the requisite skills for diagnosis or screening tools to make this determination for the study population.

### Solution

Because of the neurobiological differences among the dementia subtypes, we recommend confirming an ADRD diagnosis *via* use of the DSM-5 or National Institute of Neurological and Communicative Disorders and Stroke and the Alzheimer's Disease and Related Disorders Association (NINCDS-ADRDA) (29, 30). Implementing carefully designed diagnostic procedures will greatly increase the interpretability of study findings since the population of people living with various types of ADRD have specific diagnostic criteria (e.g., Alzheimer's disease, vascular dementia, Lewy body dementia) relative to a more general diagnosis of “dementia.” Irrespective of the selected population of interest with an Alzheimer's disease or general dementia diagnosis, we recommend performing an initial cognitive test such as the Mini-Mental State Exam (MMSE) (31), the Self-Administered Gerocognitive Exam (SAGE) (32) or the Montreal Cognitive Assessment (MoCA) (33) to screen for dementia or cognitive decline while also providing initial insight as to the severity of cognitive impairment in a potential participant.

It is important to note that many data collection instruments such as the MMSE show varying reliability and validity depending on the cut-off score chosen for the cognitive screening test used (34). For example, our previous work has demonstrated that an MMSE cut-off score of 12 has proved successful in providing responses basic pain questions such as “are you in pain now” and “please tell me your pain from 0 to 10” (35). However, we note that when using such instruments we provide a picture of the instrument with our explanation of the tool and we always read the instructions to the participant. To measure reliability, we assess if the respondent's ratings match the perceptual threshold paradigm. That is that the rating for “moderate pain” is higher than “mild pain” which is higher than “warmth.” If an individual's responses do not match this pattern, we assume an inability to fully understand the instructions and they are removed from subsequent analysis. In testing more than 50 people with Alzheimer's disease, we found one individual in which the pattern was inconsistent. Many tools have yet to be fully validated in moderate to severe dementia. We suggest that the research team agree to an acceptable level of cognitive impairment as an inclusion criterion, depending on the research question and population of interest and when studying moderate to more severe dementia the team must use a case by case approach in determining a participant's ability to provide meaningful responses (36). We also recommend that verification of an ADRD diagnosis be based on medical chart review by at least two qualified clinicians who specialize in the care of people with ADRD. In the absence of a diagnostic consensus, researchers can seek a third clinician's determination.

Another solution to this particular barrier involves use of a tool for non-clinicians to classify as Alzheimer's disease designation for research purposes. For example, the Foy Algorithm has been found to be as accurate as best clinical practice and has shown a predictive diagnostic value of >95% when compared to post-mortem evaluation for Alzheimer's disease (37). To complement any of the diagnostic screening tools, researchers should still use the medical record to confirm use of standard laboratory tests to screen for other possible causes of cognitive changes, such as hypothyroidism, vitamin B deficiencies, hypercalcemia, neurosyphilis, or human immunodeficiency virus (HIV).

## Pain medication and other study exclusion criteria

### Barrier

Numerous exclusion criteria are required to protect all persons involved in imaging research. Additional exclusion criteria specific to potential participants with cognitive impairment such as ADRD may be necessary to further ensure their comfort and safety as well as to address any questions the family or caregivers may have. Participants living with ADRD may be excluded or withdrawn from pain-related neuroimaging research for one or more of the following reasons: (1) the presence of a pacemaker, shunt, or any metal implants that are MRI incompatible, (2) severe spinal curvature, spinal disorder, or severe arthritis, (3) pain medication regimen, (4) inability to lay in a supine position for at least 10 min, (5) any acute or chronic condition that may impact testing, (6) claustrophobia, (7) absence of a legal guardian or authorized representative, (8) inability to travel to testing site, or (9) inability to provide consent or assent.

Ensuring the participant's safety in the MRI suite is especially critical, since patients or caregivers may not fully recall or even know the details of prior healthcare received. Further, older adults usually have a more extensive medical history, which means there is a higher likelihood of metal implants (38) such as shrapnel from a gunshot wound if the participant was former military personnel or the retention of a remnant in the body resulting from an industrial or factory-related accident. Surgical reports are a typical requirement for MRI clearance, which can be time-intensive and difficult to obtain, especially if the surgery occurred more than 10 years previously. The make and model of implants will typically be listed in such reports, as will MRI compatibility. Many orthopedic implants are conditionally allowed at 3 Tesla (3T), and an expert trained in MRI safety must review surgical reports to determine potential risks (39). Regardless of the source of the metal, determinations are usually necessary on a case-by-case basis as to whether the benefits of scanning a potentially vulnerable participant outweigh the risks.

### Solution

While some of these barriers are able to be addressed, some of them we are unable to overcome without further scientific advancements, such as if the individual has an implanted pacemaker/defibrillator or other implanted metals. A metal screening form completed by each participant or family caregiver and approved by an MRI technologist or another MRI safety expert before entering the scan room is the first step in proper screening. Researchers should review the metal screening form with the participant and caregiver before a scan to verify accuracy of responses and help adhere to MRI safety standards (40). Ideally the MRI screening should be completed in advance allowing for sufficient time to collect any necessary medical records needed for clearance such as surgical reports or implant device information. Asking about surgeries or implants again after the screening form review sometimes further elicits pertinent information (41). One solution to missing surgical records would involve the use of a computed tomography (CT) or an X-ray to determine placement of suspected implants (40). However, these procedures require additional consenting and explanation of the risks of exposure to these additional scans.

We recommend that a good rule-of-thumb is to assume exclusion of participants with implants in areas of neurological or cardiovascular sensitivity, such as aneurysm clips and pacemakers (40, 42). It is worth noting that some of these devices may be safe at 3T and the risk benefit must be carefully determined while also following local imaging research guidelines. In addition, dental work can present problems. Dentures as well as any other unfixed appliance, implants, or accessory should be removed prior to the scan. Permanent dental work and fillings are not usually a MRI safety concern, but they may cause undesirable artifacts in the resulting brain scan data if there is a history of extensive work (43, 44).

Many resources can assist in the resolution of questions about the MRI compatibility of a variety of medical implants. For example, MagResources and MRI safety (Reference Manual for Magnetic Resonance Safety, Implants, and Devices) are internet accessible databases that contain medical implant safety information (45, 46). If there is uncertainty with regard to a potential implanted medical device or hardware, the participant should be excluded from the MRI study.

Additional barriers identified may be also be challenging to overcome. For example severe spinal curvature, spinal disorder, severe arthritis, or other acute or chronic condition that makes it impossible or uncomfortable for a participant to lay in a supine position for at least 10 min in a MRI scanner due to pain will prohibit them from participating. Along with this, a pain medication regimen that include daily use of opioid analgesics will lead to confounds in experimental pain responses. Ethical considerations regarding an individual's need for pain relief must be prioritized over the need for research if the study design requires individuals to be free of pain medication during the time of testing. If the condition is acute, the researcher may allow the needed time for the condition to fully resolve prior to participant enrollment. The degree of claustrophobia that leads to the inability to remain in the MRI scanner prohibits study participation. Additionally, the absence of a legal guardian or authorized representative for consenting a person living with ADRD also prohibits participation. Researchers must meticulously follow the guidelines put forth by their governing IRB. The inability to provide consent or assent is addressed in the Section Capacity to consent. Solutions to the inability to travel to testing site are addressed in the Section Community engagement.

## Participant and caregiver burden

### Barrier

Extensive and detailed study requirements can result in the withdrawal of any participant from a research project. With regard to the ADRD population, such requirements may pose significant (and additional) burdens to the participant and caregiver and thus result in reluctance to participate.

### Solution

When possible, we recommend that as many of the study protocols (e.g., consenting, enrollment, short-form screening measures, or questionnaires) be carried out in the participant's home or *via* video conferencing as is feasible. This may substantially reduce participant burden without sacrificing data validity or fidelity. We further recommend that researchers reduce participant burden in relation to the degree of cognitive impairment. Based on an imaging protocol length used by ADNI (47), in-scanner sequences should be planned for <1 h, with no individual sequence lasting longer than about 5 min. Furthermore, since anxiety is known to impact brain activity during functional MRI (fMRI) (48, 49), it may be helpful to use a screening tool such as the State-Trait Anxiety Inventory–Short Form (STAI-SF) (50–52) to assess a participant's current level of anxiety before and after fMRI procedures. We have found that inviting the participant's caregiver to experience any procedure planned for the fMRI (e.g., task) will increase participant and caregiver comfort with study procedures. In our current study on Alzheimer's disease and pain (see Section Acknowledgments), we invite caregivers to accompany study participants to all study visits regardless of a participant's individual capacity. During the consenting and enrollment procedures, the caregiver are provided time to review study instruments and devices and to experience anything their loved one would be asked as a study participant. Caregivers are compensated for their time, which was applied whether or not the caregiver was involved in study procedures beyond surrogate consenting. Additionally, the use of mock scanners allows participants to experience a simulated MRI environment safely while the study team can observe for indications that the individual may not be a match for MRI procedures (excessive movement, inability to follow commands, etc.).

In addition, when approaching caregivers and people living with ADRD it is important to clarify if the study has a therapeutic intent or not. Many people participate in research with the hopes of some clinical condition possibly improving such as participation is phased clinical trials. However, much research, including MRI research, is mechanistic in nature and may not provide a direct benefit to the participant. In these cases, carefully explaining how these studies may help to inform future interventions or clinical trials typically helps maintain interest in non-therapeutic studies and also clarify benefit/burden risk for participants.

## Communication obstacles

### Barrier

Communication issues frequently pose barriers when scanning ADRD adults, which may include lessened auditory or visual acuity (53, 54) or difficulties expressing oneself (speech and language) as well as comprehension as a symptom of disease or associated with older age (55). These may present a problem not only in preparing the participant for an fMRI, but also with in-scanner tasks that require listening or reading. Hearing aids and eyeglasses are generally not MRI compatible. Expressive difficulties create further safety concerns since the participant may not be able to indicate pain, discomfort, or a desire to be removed from the scanner because of fatigue or claustrophobia (56). Further, because MRI sequences are often loud, a participant's verbalization through the scanner's intercom system may not be discernable over the noise (39).

### Solution

Provision of sensory support can assist with communication challenges. Eyeglass prescriptions may be approximated with the help of plastic goggles with lens inserts (57). Additionally, a participant's hearing may be augmented by using headphones inside of the scanner (58). Thorough observation and documentation help to verify that a participant is safe and appropriate for scanning without relying on their sometimes reduced communicative abilities. We recommend that MRI technologists, caregivers, and research staff pay special attention to the participant's needs and provide opportunities to check on everyone *via* a 2-way communication system or in-person during breaks in the MRI study.

## Relationship with MRI facility

### Barrier

Competing relationships can emerge between a research group and others collaborating within areas of the same institution (59). Scanners are often used for a number of clinical and research activities and tend to be busy during day-time hours. Participants with diminished cognition may require additional time to conduct a quality scan than what is traditionally allocated for scans of cognitive intact individuals. Coordinated assistance from healthcare providers with multiple roles, ranging from physicians and nurse practitioners to imaging staff to nurses, is crucial to study success. MRI scheduling may be mediated by a third party, while technologists at the imaging site need to be available for the entire duration of a scan. Efficient scheduling and clear communication are critical to keep all departments apprised of incoming participants and avoid creating dissatisfaction with impromptu or disorganized service. Such delays can cause anxiety or frustration for study participants and possibly also their caregivers, another potential retention risk.

### Solution

Research teams should allocate extra time for a participant to arrive for a study visit and complete pre-scan activities (questionnaires, restroom visit) without undue stress, as this greatly facilitates successful data collection. Extra time also allows a researcher to comfortably situate a participant in the scanner to minimize potential for in-scanner motion due to discomfort (60). If scanning procedures are usually scheduled for 1 h, the investigative team should consider budgeting approximately 1.5 h of scanner time. We believe that a research team that carefully considers these issues will likely experience a more positive rapport with participants and personnel at the research imaging center.

## Implications of the COVID-19 pandemic for recruitment

### Barrier

If recruitment of participants in ADRD studies was difficult prior to the COVID-19 pandemic, the emergence of this global health threat in March of 2020 with higher rates of morbidity and mortality among the older adults (61, 62) makes all aspects of study recruitment even more challenging. States and municipalities in the United States initiated a range of infection control procedures, including lockdowns, social distancing measures, travel restrictions, and institutional restrictions or closures (especially nursing homes and long-term care sites) (63) that had a deleterious impact on clinical research activities and ongoing recruitment initiatives. In addition, in our experience, the burden of clinical care and diagnosis in hospitals and associated imaging facilities was shifted to COVID patients, which meant decreased schedule access and technician availability. While the advent of several vaccines starting in the spring of 2021 eventually resulted in the easing of some restrictions (64) as of this writing, the pandemic continues to evolve and thus poses a continuing hazard to older adults who most commonly qualify as study participants living with ADRD. As noted by Brown et al., these individuals are “among the most vulnerable persons in society, depending on family or professional caregivers for their day to day survival,” (65) and this situation has only worsened during the pandemic.

### Solution

The impact of COVID-19 will significantly impact recruitment processes and study protocols in ADRD research. Simultaneously, researchers who have developed community partnerships with stakeholders in relevant settings (senior centers, assisted living facilities, support groups, etc.) are at an advantage since they will be able to more easily target social media, email list servs, and other online platforms for publicizing and promoting study participation. For example, some centers may recommend setting up a virtual town hall series for clinical sites, community-based organizations, or advocacy organizations to permit an interactive venue by which participants and caregivers can learn more and pose questions about the project. Traditional print materials, such as posters and flyers, can be made “contactless” or “touchless” *via* the inclusion of a QR (Quick Response) code, which when scanned *via* a mobile device such as a phone or tablet provides direct access to a website with more information about how to join a study (66).

Principal investigators should minimize face-to-face contact and employ protective gear, handwashing, and social distancing in all interactions with study participants, or explore ways in which assessment and monitoring activities might be conducted remotely, *via* email or digital platforms such as Facetime or REDCap (Research Electronic Data Capture) (66). Data collection requiring in-person visits such as neuroimaging would necessitate new consultations with the ADRD participant or caregiver so that both are fully aware of increased exposure risks, as well as the continuing involvement of IRBs to determine the risk/benefit ratio in these circumstances (67). In this regard, the development of trusted relationships between the research team and community ambassadors becomes doubly important. This may include screening for COVID-19 symptoms on study visit day and voluntary reporting of research staff, participant, and caregiver vaccination or antibody status.

As Brown et al. commented, “ADRD has become one of the most active areas of both basic and clinical research, attracting major industry, government, and philanthropic funding in the Western world… [a]brupt interruption of all these research studies would not only jeopardize a crucial investment society has already made, but it could also have long-lasting consequences for the field of ADRD research” (65). However, not all of these consequences might be negative; the pandemic may well provide researchers with an impetus to innovate new remote methods for data collection as well as explore new applications of existing technologies while minimizing risk to study participants. For example, using social media platforms, local neighborhood news flyers, and public transportation systems are measures that can reach an increased number of potential participants. Table 1 summarizes the barriers and proposed solutions presented in this paper.

**Table 1:** Barriers and solutions for recruiting persons living with ADRD for pain-related neuroimaging studies.

**Barrier**	**Solution**
1. Community engagement	•Seek, develop, and maintain community partnerships •Address culturally-specific barriers such as trust due to historical racial traumas •Provide appropriate financial compensation for time commitment •Include study personnel with expertise/focus on recruitment
2. Capacity to consent	•Use family caregiver/surrogate decision maker as needed •Use assent form in addition to consent form •Use established measures to determine capacity to consent such as MacCAT-CR and UBACC •Use brief 3-item Capacity to Consent questionnaires
3. Dementia diagnostic criteria	•Consider neurobiological differences in dementia subtypes •Identify selective cognitive impairment inclusion criteria using standardized instruments such as MMSE •Use medical chart diagnostic criteria •Consider use of tool such as the Foy Algorithm when clinical diagnosis is not accessible
4. Pain medication and other study exclusion criteria	•Prioritize MRI safety through meticulous screening and accessing of historical medical records •Provide opportunity to view and experience equipment in advance if claustrophobic •Consider that many criteria cannot be overcome e.g., inability to lie supine for at least 10 min and need for daily opioid medications due to pain •Consult with governing IRB early and often
5. Participant and caregiver burden	•Be flexible with study location e.g., virtual or in-home consent and questionnaire completion, if possible •Use shortform versions of questionnaires, if available •Use shortest possible in-scanner sequences •Allow opportunity to preview study questionnaires in advance, as appropriate to study design •Provide clarity on therapeutic or non-therapeutic study intent
6. Communication concerns	•Conduct frequent in-person and 2-way communication system check-ins with MRI staff and caregiver and consistent ability to visualize participant •Provide needed sensory support e.g., plastic goggles (with lens inserts) and headphones
7. Relationships with neuroimaging facilities	•Allocate extra time for participant arrival •Include additional time for MRI completion
8. COVID-19 pandemic implications	•Anticipate pandemic-related recruitment delays •Consider infection prevention protocol changes and delays •Use technology to limit face-to-face contact as much as possible

## Conclusions

In summary, pain-related neuroimaging studies are time-intensive and sometimes tedious but are of ever-increasing importance since they can substantially impact what we understand about disease processes. For pain-related neuroimaging studies that include vulnerable populations, such as those living with ADRD, a plethora of barriers exists that renders recruitment a daunting task. However, as stated in the Belmont Report, all individuals deserve and should be included in research to advance the science and improve outcomes, especially vulnerable populations (1). Thus the research team is required to be accommodating and attentive to the needs of participants, their families, and other loved ones, including cultural considerations. Attending to the guidelines provided here—community recruitment, capacity to consent, dementia diagnostic criteria, pain medication and other exclusion criteria, patient and caregiver burden, communication concerns, relationship with MRI facility, and implications of the COVID-19 pandemic for recruitment—can help minimize these barriers.

The impact made by demonstrating a high level of competency and caring by research staff is vital to ensuring potential participants' desire to partake in the research. Participants who have an unpleasant experience during a study are likely to speak poorly about the research to their friends and family, and as noted above, since research participants are often acquainted with like-minded or similarly afflicted (in the case of ADRD) individuals, this may lead to the loss of multiple potential participants.

Incorporating practices as outlined in this paper into pain-related neuroimaging studies investigating ADRD is a prudent measure to increase recruitment and research output. The time invested into maintaining community and institutional relationships, as well as strict adherence to research protocols will yield a high return on investments *via* participant satisfaction and cleaner data collection. These necessary measures will influence future research participation within good ethical practice guidelines contributing to a greater understanding of ADRD through neuroimaging and subsequent improved health outcomes.

## Author contributions

All authors listed have made a substantial, direct, and intellectual contribution to the work and approved it for publication.

## Funding

We would like to acknowledge the following financial support: NIH/NIA R01AG059861, R01AG061325, R21AG045735, R21AG049332, K23AG046379, 3R01AG059861-02S1, The Mayday Fund, and The John Hartford and Atlantic Philanthropies Foundation.

## Conflict of interest

The authors declare that the research was conducted in the absence of any commercial or financial relationships that could be construed as a potential conflict of interest.

## Publisher's note

All claims expressed in this article are solely those of the authors and do not necessarily represent those of their affiliated organizations, or those of the publisher, the editors and the reviewers. Any product that may be evaluated in this article, or claim that may be made by its manufacturer, is not guaranteed or endorsed by the publisher.
